# Optional part-time and longer GP training modules in GP practices associated with more trainees becoming GPs – a cohort study in Switzerland

**DOI:** 10.1186/s12875-017-0706-1

**Published:** 2018-01-05

**Authors:** Lara Studerus, Regina Ahrens, Christian Häuptle, Adrian Goeldlin, Sven Streit

**Affiliations:** 1 Institute of Primary Health Care Bern (BIHAM), University of Bern, Gesellschaftsstrasse 49, 3012 Bern, Switzerland; 2 Foundation to Promote Training in General Practice (WHM), Bern, Switzerland

**Keywords:** Primary care, GP shortage, GP training, Residency

## Abstract

**Background:**

Switzerland, like many other countries, has a shortage of General Practitioners (GPs). Optional GP training modules in GP practices were offered during the at least 5-year GP training program to increase student and trainee interest in becoming a GP. The training modules had not yet been evaluated. We determined how many Swiss GP trainees became practicing GPs after they completed optional training modules, and if longer modules were associated with higher rates of GP specialization.

**Methods:**

In this population-based cohort study, we included GP trainees who chose an optional GP training module in GP practice, provided by the Foundation to Promote Training in General Practice (WHM) between 2006 and 2015. GP trainees were invited to complete an online survey to assess the primary outcome (becoming a practicing GP by 2016). Data on non-responders was collected via an internet search. We calculated univariate time-to-event curves to become a practicing GP, stratified by trainee’s gender, length, part-time training, and number of years after graduation until training modules were completed. We used a multivariate model to adjust for characteristics of participants, training, and satisfaction with training modules.

**Results:**

We assessed primary outcome for 351 (92.1%) of 381 former GP trainees who participated in a WHM program between 2006 and 2015. Of these 218 (57%) were practicing GPs by 2016. When focusing on the trainees who had completed training between 2006 and 2010, the rate of practicing GPs was even 73%. Longer (*p* = 0.018) and part-time training modules (*p* = 0.003) were associated with higher rates of being a practicing GP. Most (81%) practicing GPs thought their optional GP training module was (very) important in their choice of specialty.

**Conclusion:**

GP trainees who spent more time training in a GP practice, or who trained part-time were more likely to become practicing GPs. Most (80%) rated their training module as (very) important in their choice of career, highlighting that these modules effectively encourage the interests of those already inclined towards the GP specialty. Longer GP training modules and more opportunities for part-time training may attract and retain more interested trainees, and possibly increase the number of practicing GPs.

**Electronic supplementary material:**

The online version of this article (10.1186/s12875-017-0706-1) contains supplementary material, which is available to authorized users.

## Background

Western countries face a GP shortage [1–5] because aging populations require more care and place more pressure on health care, [6, 7] and because not enough doctors specialize in general practice [2, 3, 5]. To address the growing shortage of GPs, many highly-developed countries have set up training programs for GPs and designed them to raise the attractiveness of the profession [8–10]. In 2011, the Organisation for Economic Co-operation and Development (OECD) determined that GP-centred health systems needed 1 GP per 1000 inhabitants to function well [11]. Switzerland, for example, has a shortage of 2000 GPs, which will worsen when 60% of GPs retire by 2025; without an increase in the number of young physicians who choose primary care, Switzerland will be about 5000 GPs short by 2025 [12]. Every year, 900 Swiss medical students graduate, but only 10–20% intend to become GPs [12, 13]. Though many papers evaluate how effectively programs train GPs [14, 15], far fewer ask if GP training programs successfully increase the number of trainees who decide to become GPs [9].

In Switzerland, as in many other Western nations, the GP specialty carries less prestige than many other specialties. In the academy, most specialties are held in higher regard than primary medicine [13, 16, 17]. Medical students and trainees are often asked by members of the public whether they will become a GP or choose a specialty, as if the former were not a specialty [18, 19]. In general, GPs work long hours [20] , and work in less attractive places [19]. Students may also be intimidated by the breadth of knowledge required, or feel they do not have the personality traits needed by a good GP (“bedside manner”) [19, 21]. Academic training programs have increasingly emphasized the importance of primary care and GP training, which may have begun to shift impressions about the attractiveness of the specialty, but they do not yet attract enough young doctors to address the shortage [12].

Over the last 15 years, Switzerland has attempted to raise interest in becoming a GP among medical students and recently graduated physicians. A 2011 study found only 38% of the Swiss trainees who said they wanted to become a GP were working as GPs within 8 years of finishing medical school [22]. To address GP shortages, we must increase this percentage both by creating more interest in the profession, and encouraging those who are already interested to follow through and go into practice [12]. To increase the number of doctors, the state has funded many new places at medical schools (undergraduate programs). Medical schools offer combined lectures (GP and specialist), one-on-one GP tutoring during basic medical education, and mandatory clerkships in primary care [23, 24]. Switzerland has also invested heavily in postgraduate training for GPs, which is when young doctors specialize. Traditionally, Swiss postgraduate GP training lasts five years, two of which must be spent training in internal medicine. Swiss trainees must also meet other requirements, such as passing a national exam and completing required courses [25]. The state has raised the profile of primary care at the major universities by opening new Institutes for Primary Cares [23] that take responsibility for training and providing continuing education courses for GPs [8]. These Institutes have developed programs and curricula [8, 23, 26]. One such program is an expanded GP training module (Praxis Assistenz) developed and maintained by the Foundation for the Promotion of Education in Primary Care (WHM). The program places trainees in GP offices for 6–12 months [27].

The Praxis Assistenz program is available to trainees both directly through the WHM, and through cantonal programs with which WHM works closely [26]. Currently, any trainee who fulfils mandatory requirements can apply to be board-certified as a GP, without ever having worked in a GP practice [25]. Praxis Assistenz was designed to give trainees practical experience in a GP office, exposing them to the day-to-day operations of a real-world practice. Residency in a family practice is one of the most important incentives for starting a family practice, followed closely by a personal relationship with a family physician [19]. The designers hoped the program would create a supportive mentoring environment in which trainees would learn the skills GPs need, and reduce their prejudices against the specialty. Though the program has been in place and expanded for over 15 years it, like most other post-graduate GP training programs, has not yet been evaluated for effectiveness. Two meta-analyses of the effectiveness of interventions intended to increase the proportion of student who choose primary care were conducted ten years apart. As the authors of both meta-analysis note, longitudinal clerkships were the only intervention consistently found to be effective. Both meta-analyses noted issues with the quality and design of the evaluation studies they analysed, and named the largest problems: confounding due to absence of adjusted statistical analyses; risk of selection bias; risk of recall bias; small sample size; and absence of a control group [10, 28]. Some of these quality problems are very difficult to avoid, given the conditions in which interventions are developed and tested. For example, there is no standard way to sample the interests and intentions of students, which can change over time, and be remembered differently. In Switzerland, as in some other countries, students or young doctors may express an interest in specializing, but do not need to make a commitment. Control groups are difficult to set up when programs are voluntary, because electing to go into a program already reflects a bias, and impossible to set up when programs are mandatory. Programs often start small, without resources for careful evaluation, and grow quickly and organically, influenced by both physician and trainee needs, and political pressures [29]. Before and after comparisons may be the closest we can get to approximating a control group. For example, we know that 15% of all recent graduates of Swiss medical schools expressed a wish to become GPs. Of this subgroup, only 38% had become GPs eight years later [22]. It may be that the best measure we can have of the effectiveness of training programs is the increase in the rate of participants who do become GPs, over the average rate in Switzerland.

We conducted a population-based cohort study based on an internet survey and previously recorded data to determine how many GP trainees in a nationwide GP training module program became practicing GPs, and if longer training (6 months in a practice vs. 12 months) increased the number of trainees who become GPs. A secondary goal was to identify other training-related factors that might predict whether a trainee would become a GP.

## Methods

### Study design

**Fig. 1:**
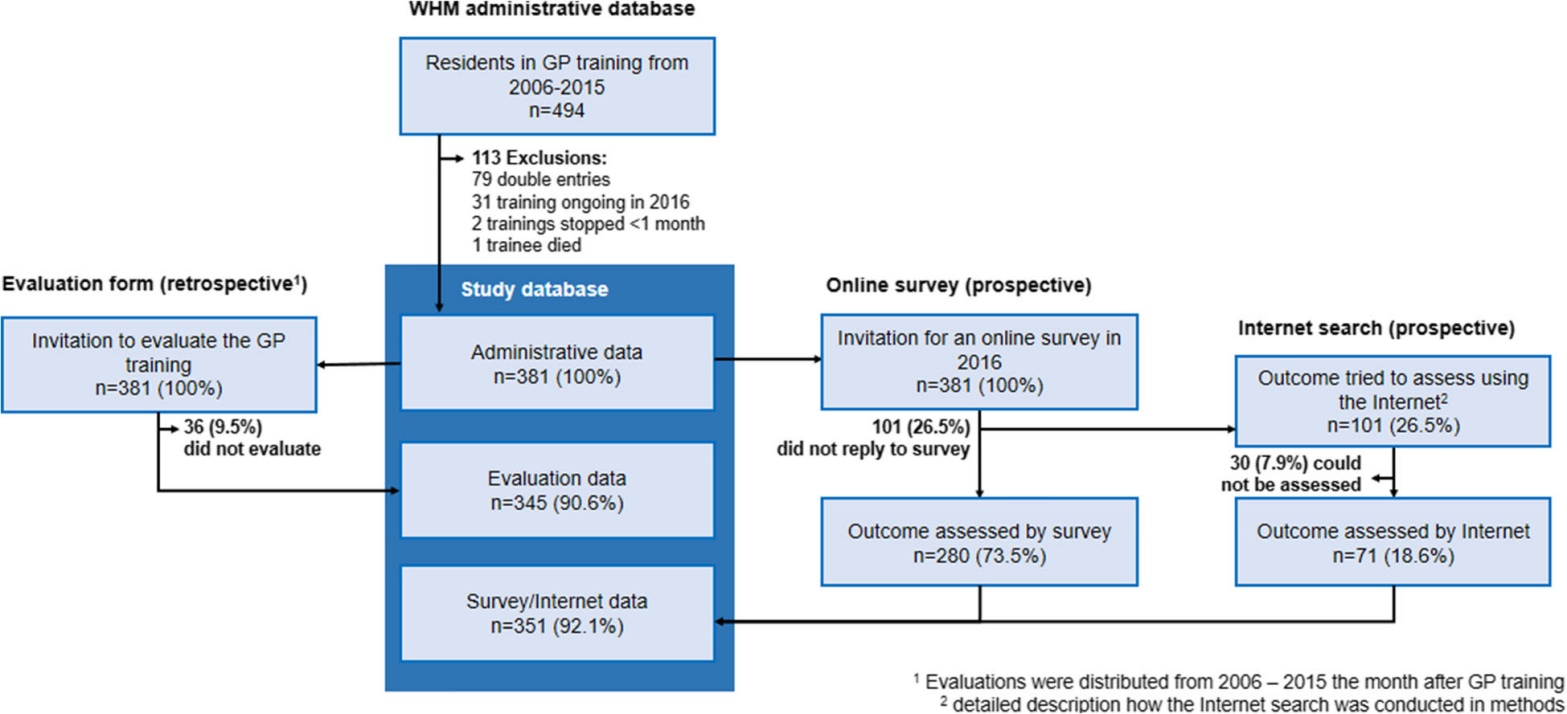
Flowchart and sources of data. We used an administrative data base of WHM foundation after excluding data entering errors. To assess exposure variables, we used records of evaluation forms replied by >90.6% of all GP trainees after completion of the GP training module in GP training. To assess the outcome to become or not become practicing GP by 2016 we used two prospective approaches: first, an online survey and second, an internet search among non-responders to the survey. Blue underlined are the data we used for this study

In this population-based cohort study, we assessed our outcome (becoming practicing GP by 2016) with a survey and an internet search to identify non-responders. The cohort was all participants in the WHM training program between 2006 and 2015 (*n* = 381). We used retrospective data from the WHM administrative database, and supplemented it with an online survey administered to training program participants in 2016. More data on the exposure (GP training module) and trainee’s satisfaction with the module was prospectively collected from 2006 to 2015 (Fig. 1).

### Outcome

Our primary outcome was the working status of former GP trainees (practicing GP by 2016: yes/no). Our secondary outcome was common program-related parameters that favoured or deterred trainees.

### Training program

WHM GP training places trainees at a GP office, where they work under the supervision of a GP (trainer). All GP trainers attended a two-day course on how to supervise GP trainees. WHM and GP trainers pay GP trainee salaries [27]. GP trainees must declare their interest in becoming a GP. In Switzerland, trainees can change their career path during or even after their GP training.

GP training modules are now also organized at a regional level [26]; WHM often administers candidates and salaries for these programs. Thus, WHM is responsible for most GP trainees. More details about the WHM program can be found on www.whm-fmf.ch.

### Participants

Between 1998, when WHM offered the first GP training module, and 2015, 809 GP trainees completed the program. We included all trainees who registered for a GP training module in GP offices with WHM in the last 10 years (from 2006 to 2015, Fig. 1). We chose this period because, throughout this period, the evaluation forms were the same. We had 494 trainee records. Exclusion criteria were 1) those who had not completed training; and, 2) those who dropped out of training in under a month. No trainee was counted more than once, because we merged the records of trainees who had completed multiple modules and summed their hours.

### Data sources

WHM registers all trainings in a database that contains administrative data about the trainees and the trainers, and the evaluation forms trainees and trainers complete when they have finished a training.

In January, 2016, we sent an email invitation to all trainees in the WHM database (2006–2015) to assess our primary outcome. We invited them to complete a short online survey in either German or French (see Additional files 1 and 2). Participants were asked for their current working status. Practicing GPs were then asked when they started practicing, and how much influence their GP training module had on their decision (5-point Likert scale). The other questions were demographic. If they did not respond, they were sent two email reminders (2 weeks and 4 weeks after the initial invitation).

To assess our primary outcome for trainees who did not respond after two reminders, we first searched the National Registry of Medical Professions in Switzerland (www.medreg.admin.ch), which prospectively records all graduating medical students in Switzerland. The register is public and notes whether and when trainees were board-certified as GPs. However, in Switzerland, a board-certified GP might not be in practice as a GP (e.g., they could be doing research, positioned abroad, working for an insurance company, etc.). If we could not locate them, we searched professional directories, including www.doktor.ch, www.doctorfmh.ch, social media websites, including www.linkedin.com, www.facebook.com, and www.google.ch to find each trainees’ current working status. If we could not determine current working status, we categorized these trainees as “could not be assessed” and excluded them from the analysis. Based on previous experience with this kind of internet search, we expected that <10% of trainees would be excluded for this reason.

We used data from WHM database to capture GP trainees and GP trainer characteristics. For GP trainees, we collected data on gender, age at time of GP training, the years GP training started and ended, months of training, percent of training if part-time (e.g. 50% = 2.5 days/week), and how long after graduation the GP entered the program. We also stratified length of GP training module based on length of training (adding up part-time training so it was a percentage of full-time; for example, 2 months of 50% training were counted as 1 month of full-time training). We stratified by three periods because, in Switzerland, stakeholders want to know if length of training (<6, >6, or >9 months) has an effect on a trainee’s decision to become GPs.

For GP trainers, we collected data from WHM, including how old the trainers were when they taught the module, gender, and type of practice (e.g., solo or group practice). We used the postal code of their GP practice to categorize GP practices by population density, which we calculated with public population census data.

To collect data on satisfaction of GP trainees with the GP practice where they trained, we analysed the evaluation forms that WHM routinely collects immediately after the GP completes the training module. However, since the evaluation form is very long (79 questions) we a priori selected a sample of questions to analyse. Limiting our sample helped us avoid methodological problems caused by overfitting. If we had included all the questions, and used a significance level of 5%, one in twenty associations we identified would have been a statistical artefact. We thus invited a panel of experts to choose questions they though most likely to be associated with the exposure (longer vs. shorter GP training) and the outcome (becoming a practicing GP by 2016). We decided in advance that we would include questions that at least 5 of 6 experts had selected. Four out of the original 79 questions met that criteria: 1) overall satisfaction with the training module; 2) quality of supervision by GP trainer; 3) ability to acquire perceived competencies during GP training module; and, 4) trainee’s opinion of how well the GP trainer taught.

### Statistical analyses

We described basic characteristics of GP trainees, GP training modules, GP trainer, and satisfaction with GP training module, both for GP trainees who became practicing GPs and those who did not. We used the chi square test to compare categorical data, and the t-test or non-parametric Wilcoxon ranksum test, where appropriate, to compare continuous data.

We calculated univariate time-to-event curves to become practicing GP and stratified the data by GP trainee’s gender, part-time training (yes/no), length of GP training module (<6/>6 months), and whether they were trained before or after the fourth year of GP training. The curves were constructed with the Kaplan-Meier method, and compared using a log-rank test. We defined time-to-event as starting the year GP training module was completed and ending the year the trainee went into practice as a GP. If time data for individuals was missing, we calculated the mid-point between the end of the GP training module and the time the subject completed the questionnaire. For all cofactors, we calculated hazard ratios (HRs) to become practicing GPs and corresponding 95% confidence intervals (CI). We calculated both univariate and multivariate HRs with Cox proportional hazard models. In a multivariate model, we further adjusted for gender of both GP trainee and GP trainer, year of training, part-time training, duration of training (calculated based on full-time training, stratified into <6 months, 6–9 months, or >9 months), dates of training (stratified into three groups: 2006–2008, 2009–2012, and 2013–2015), solo practice, and the four a priori questions experts had chosen from the evaluation form. We drew forest plots that used random effects models to visualize the associations of the selected cofactors using HRs.

We considered a two-sided *p*-value of 0.05 to be statistically significant. We performed all analyses with STATA version 14.2 (Stata Corp, College Station, TX, USA).

## Results

We began with 494 records for GP trainees who finished a GP training module in GP practices between 2006 and 2015. Based on our criteria, we excluded 79 (16.0%) double entries; 31 (6.3%) records were for ongoing training; 2 (0.4%) trainees dropped out in less than a month, and 1 (0.2%) trainee had died. (Fig. 1).

Our final sample included 381 trainees, to whom we sent the email invitation to participate in the online survey. Of these, 280 (73.5%) replied to the online survey. Of non-responders (*n* = 101), we identified 71 (70.2%) via internet search. (LS did the internet search and SS checked a random sample of 10 individuals and reached consensus on all 10 classifications.) We had to exclude only 30 (7.9%) trainees. We thus assessed primary outcome in 92.1% of all the trainees we included. Non-responders were similar to responders in gender (*p* = 0.69) and age (*p* = 0.10).

**Fig. 2:**
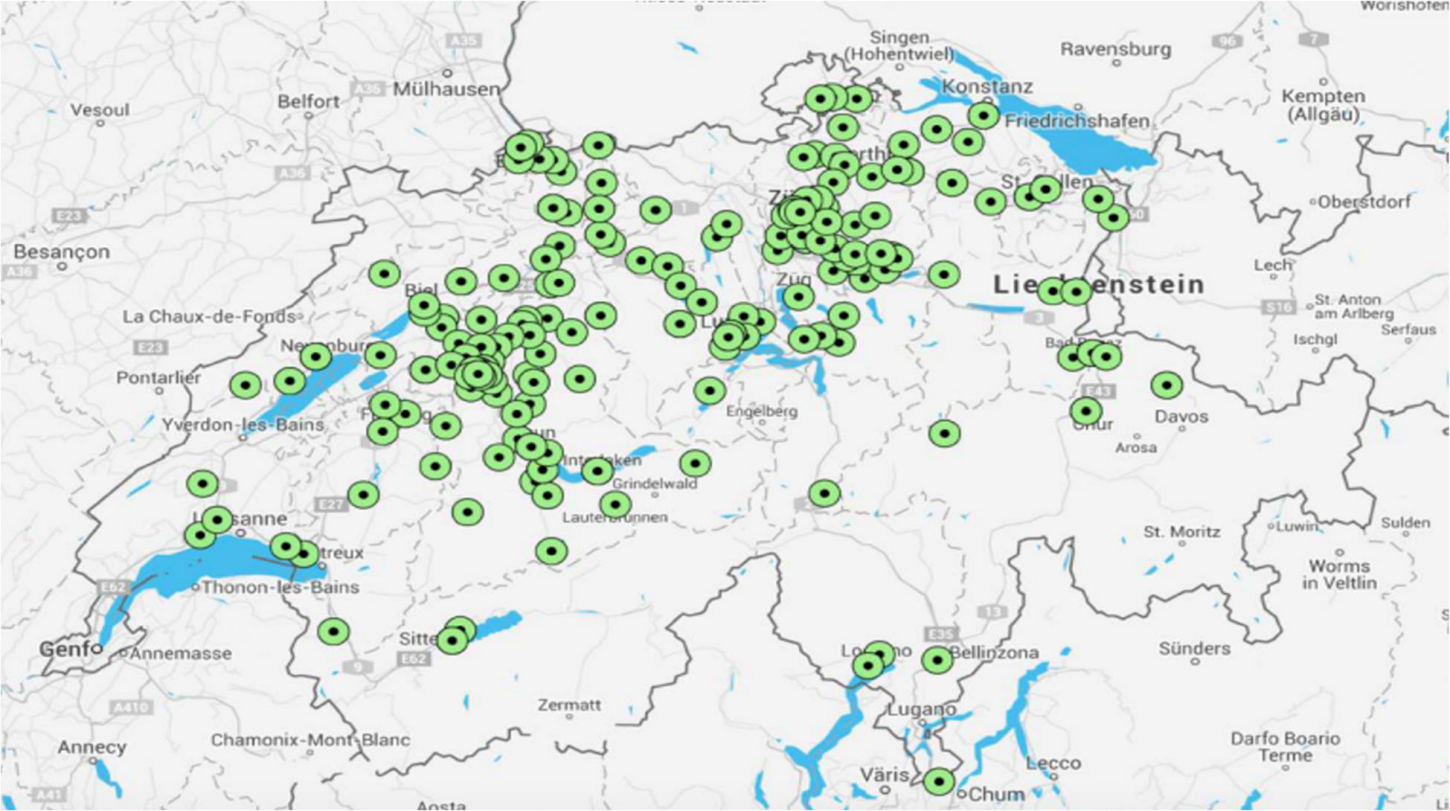
New GPs after GP training module: places of working. Places in Switzerland, where new GPs started to work after GP training module (every green dot represents 1 new GP). Data based on ZIP codes. Graphic made by using Google Maps and BatchGeo LLC (http://batchgeo.com)

By 2016, 57.2% (95%CI 52.1–62.2%) trainees had become practicing GPs. However, when we restricted our analysis to 2006–2010, the percentage increased to 73.6% (95%CI 66.4–79.9%). On a per capita basis, new GP practices were distributed almost evenly across the inhabited area of Switzerland. (Fig. 2). A large majority of trainees (81%, 95%CI 75%–87%) thought their GP training module in GP practice had positively influenced (very important/important) their choice to become practicing GPs. Table 1 describes the basic characteristics of GP trainees, the GP training module in GP practice and their satisfaction with the training module.

**Table 1:** Characteristics of GP trainees and training module, GP trainers, and GP trainee satisfaction with GP training module in GP practice, stratified by whether former trainees became GPs by 2016

GP trainee and GP training module	Overall n = 381	GP^a^ *n* = 218 (57%)	Non-GP^a^ *n* = 133 (35%)	*p*-value
Female, n (%)	233 (66.4)	142 (65.1)	91 (68.4)	0.53
Age, mean (SD)	33.7 (4.1)	34.5 (4.1)	32.4 (3.6)	<0.001
Year of graduation from medical school, mean, rounded (SD)	2005 (4)	2003 (4)	2007 (4)	<0.001
Time range of training module, n (% per column)				<0.001
2006–2008	103 (29.3)	81 (37.2)	22 (16.5)	
2009–2012	135 (38.5)	94 (43.1)	41 (30.8)	
2013–2015	113 (32.2)	43 (19.7)	70 (52.6)	
Average number of years between graduation and start of GP training (SD)	4.2 (1.9)	4.5 (1.9)	3.8 (1.8)	<0.001
Length of training module in months (calculated based on 100%), n (% per column)				0.03
< 6 months	81 (23.1)	48 (22.0)	33 (24.8)	
6–9 months	222 (63.3)	132 (60.6)	90 (67.7)	
9–12 or more months	48 (13.7)	38 (17.4)	10 (7.5)	
GP trainer characteristics				
Female, n (%)	20 (5.9)	12 (5.7)	8 (6.1)	0.90
Age, mean (SD)	54.7 (7.6)	54.6 (7.6)	54.8 (7.5)	0.83
Office type, n (% per column)				0.60
Solo practice	151 (46.6)	95 (47.5)	56 (45.2)	
Dual practice	71 (21.9)	46 (23.0)	25 (20.2)	
Group practice	102 (31.5)	59 (29.5)	43 (34.7)	
Tertile of population^b^ near GP office (calculated by zip code) n (% per column)				0.43
< 4000 inhabitants	118 (34.3)	78 (36.5)	40 (30.8)	
4000–12,000 inhabitants	114 (33.1)	66 (30.8)	48 (36.9)	
> 12,000 inhabitants	112 (32.6)	70 (32.7)	42 (32.3)	
GP trainee satisfaction with GP training module in practice				
Overall satisfaction, scale^c^ (SD)	3.7 (0.7)	3.8 (0.6)	3.6 (0.8)	0.035
Quality of supervision by GP trainer, score^d^ (range 1–42) (SD)	31.0 (7.6)	31.6 (7.6)	30.1 (7.5)	0.093
Perceived competencies acquired during training, scale^c^ (SD)	2.9 (0.9)	3.2 (0.8)	2.5 (0.8)	<0.001
Quality of teaching by GP trainer, score^d^ (range 1–21) (SD)	18.8 (3.3)	18.9 (3.3)	18.7 (3.4)	0.74

^a^We could not asses 30 (7.9%), so the combined groups total 351 instead of 381 individuals

^b^We used ZIP codes of GP offices and linked them to 2014 census data to determine population density (source: Bundesamt für Statistik)

^c^Answers were rated 1 “not true”, 2 “mostly not true”, 3 “mostly true”, 4 “true”

^d^We assessed this with multiple questions. For analysis purposes, we summarized the answers in a single continuous score. The higher the score, the better the evaluation

Several characteristics showed statistically significant differences: GP trainees who became practicing GPs by 2016 were older (34.5 vs. 32.4 years, *p* < 0.001), graduated earlier from medical school (year 2003 vs 2007, p < 0.001), completed their GP training module later in their GP training (year 4.5 after graduation vs. 3.8, p < 0.001), and trained longer (e.g. 17.4% >9 months training vs. 7.5%, *p* = 0.03). Trainer characteristics did not seem to be associated with becoming or not becoming a GP. Trainees who became practicing GPs by 2016 were more satisfied with their training modules (score 3.8 vs. 3.6, *p* = 0.035), and felt they had gained more skills and confidence during training (score 3.2 vs. 2.5, p < 0.001) than trainees who had not become practicing GPs.

**Fig. 3:**
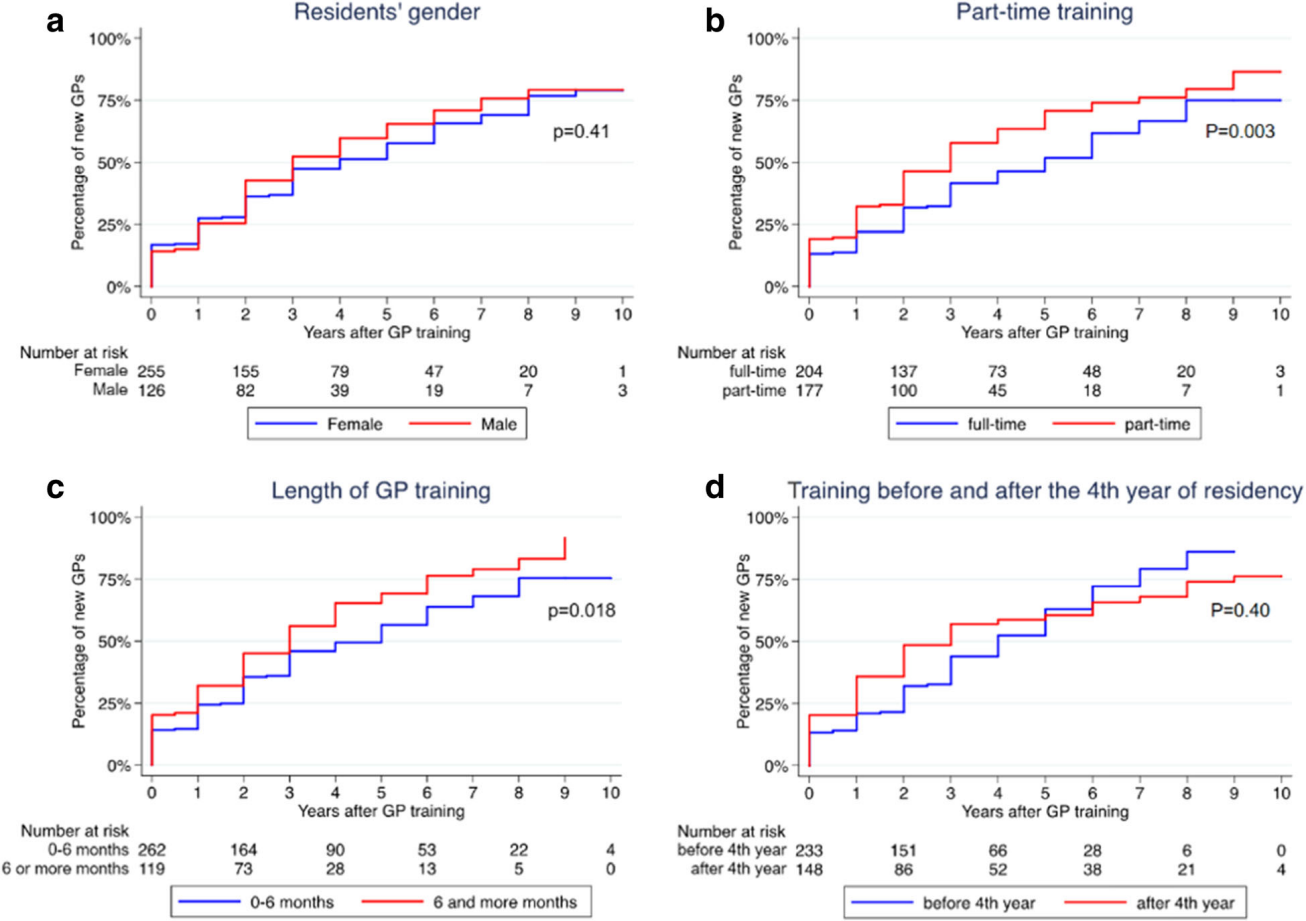
Time to become practicing GP after optional GP training modules in GP practices. Percentages of practicing General Practitioners (GPs) after an optional GP training module in GP practices using time-to-event analysis. *P*-values were calculated using log-rank tests

We calculated time-to-event curves to becoming a practicing GP after GP training, for four different subgroups (Fig. 3). Two factors shortened this time period to a statistically significantly degree: part-time training (*p* = 0.003) and training for 6 or more months (*p* = 0.018). The trainees’ gender (*p* = 0.41) and training before or after the fourth year after graduation (*p* = 0.40) did not have a significant effect on time to practice.

**Fig. 4:**
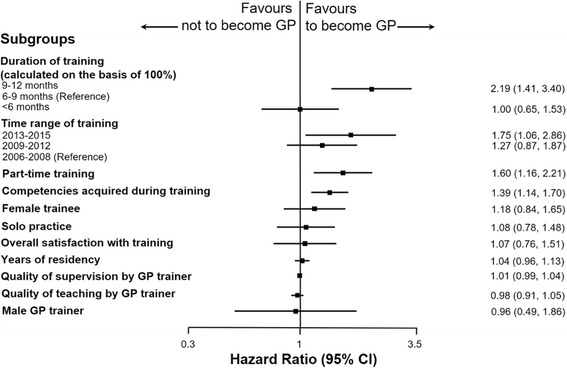
Multivariate analysis of cofactors and their association to become practicing GP. Hazard ratios (HRs) of subgroups are calculated using multivariate cox regression using a random effects models. HRs are adjusted for all cofactors shown in the figure and sorted top-down

The forest plot (Fig. 4) shows the results of our multivariate Cox model. Co-factors significantly associated with trainees becoming practicing GPs by 2016 were: 1) GP training modules of 9–12 months (HR 2.19, 95%CI 1.41, 3.40); 2) training between 2013 and 2015 (HR 1.75, 95%CI 1.06–2.86); 3) part-time GP training (HR 1.60, 95%CI 1.16–2.21); and, 4) a trainees’ belief that training helped them acquire necessary competencies (HR 1.39, 95%CI 1.14–1.70). In our model, no characteristic significantly deterred trainees from becoming practicing GPs.

## Discussion

### Summary

Of those who completed the WHM program (2006–2015), 57% were practicing GPs in 2016. The large majority of practicing GPs (4 out of 5) rated their training module as important/very important in their choice of career. We found three factors were significantly associated with choosing a GP career: 1) length of GP training module (9–12 months vs. 6 months of full-time training); 2) part-time training; and, 3) a trainee’s belief they were very competent at the end of the training module. Trainee gender, GP trainer characteristics, or whether trainees participated in the training before or after their fourth year after graduation were not significant factors.

The high percentage of trainees who become GPs suggests that the program is, overall, effective. An earlier Swiss study found that, of the trainees who declared their intention to become a GP, only 38% of them had done so 8 years later [22]. Other studies have also found that GPs think their training modules were important to their careers [9], which suggests that expanding and improving GP training programs may encourage more young doctors to become GPs. We did expect to find that trainees who became practicing GPs were likely to have spent more time in training [9, 10, 28]. We cannot tell if longer training makes someone likelier to choose the GP specialty, or if people who are already determined to choose the specialty generally decide to train for longer periods of time (reverse causality).

What we did not expect is that those who trained part-time (accrued their time over longer periods) would be more likely to become GPs. Again, we cannot determine the flow of causality. We do know that training part-time lengthens the duration of exposure, even when it does not increase training hours, and more exposure may generate more interest [9, 10]. It is also possible that part-time training allows trainees to better balance private and professional life. Research in Switzerland suggests that young doctors are more interested in working part-time, regardless of gender, and that part-time work opportunities make it easier for young doctors to choose the GP specialty [30]. Another possibility is that trainees feel more competent at the end of longer GP training, and longer training may abate their fears of not being competent or well-suited to being a GP [9, 10, 28].

Other studies found that women students and trainees are more likely to choose the GP specialty [16, 17, 31]. There were also more women in the GP training module program (66%), but there was no gender difference between trainees who became or did not become practicing GPs. It also made no difference whether trainees started their training module before or after their fourth year of GP training. We also looked at practice type, localization, gender of trainee and trainer, quality of supervision or teaching, and found no significant association with our primary outcome.

### Strengths and limitations

Our study is limited by its observational design: we can describe associations, but cannot prove causal links between GP training modules and the decision to practice as a GP because the association could run in either direction (reverse causality). However, all GP trainees who participated in the GP training module we studied had already confirmed their interest in working as GPs after training. The effect sizes we found were rather small (HR 2.2 for long training modules, and HR 1.60 for training modules in part-time), which make a causal relation less certain. Our results, however, agree with those in other studies [9, 10].

Our study also had several strengths. We compared trainees to each other, and based our analysis on data collected over a decade in the same GP training program. The response rate was high both for the evaluation survey, and our later online survey, which lowers the risk of selection bias. Analysing retrospective evaluation forms helped us avoid recall bias, and we also prospectively assessed the current working status of former GP trainees for the same cohort, giving us a clear picture of both past and present opinions of trainees about the program they completed.

### Implication for practice and research

Since most GPs who complete the program become GPs, and a large majority of these GPs rated their GP training as a very positive influence, we recommend creating more opportunities for future GPs to attend such programs. Offering GP training modules that last for more than 6 months, and giving trainees the option to train part-time program could attract trainees already interested in becoming GPs and encourage more young doctors to choose the GP specialty by exposing them to practices for a longer time. We should also investigate further to determine the flow of causality; it is essential to find out if very interested trainees choose to train longer, or if longer training programs increase interest in becoming a GP. We also suggest researchers evaluate other post-graduate programs and compare their results to ours.

## Conclusions

Of those who completed the WHM program (2006–2015), 57% were practicing GPs in 2016; 73% of those who completed the program by 2010 had become practicing GPs. GP trainees who chose trained for longer or trained part-time in a GP practice were more likely to became practicing GPs. Four of five GPs rated their training module as a very strong influence on their career choice, highlighting the importance of this GP training module. Offering more, longer, and more flexible (part-time) positions in GP training programs may increase the interest of young doctors in becoming practicing GPs.

## Additional files

Additional file 1: English version of the survey used. (PDF 180 kb)

Additional file 2: German and French version of the survey used. (PDF 266 kb)

## Abbreviations

BIHAM: Berner Institut für Hausarztmedizin (Institute of Primary Health Care Bern)
CI: Confidence Interval
DSG: Swiss Data Protection Act
GP: General Practitioner
HR: Hazard Ratio
OECD: Organization for Economic Co-operation and Development
SD: Standard Deviation
WHM: Stiftung zur Förderung der Weiterbildung in Hausarztmedizin (Foundation for the Promotion of Training in General Practice)
