# Supplementary material for: Optional part-time and longer GP training modules in GP practices associated with more trainees becoming GPs – a cohort study in Switzerland
Source: BMC Fam Pract. 2018 Jan 5;19:5. doi: 10.1186/s12875-017-0706-1 (PMC5756440; doi:10.1186/s12875-017-0706-1)
Supplement: Supplementary file 1 — English version of the survey used. (PDF 180 kb) [file 12875_2017_706_MOESM1_ESM.pdf]

Wählen Sie Ihre Sprache / Choisissez votre langue

* Wählen Sie Ihre Sprache / Choisissez votre langue

◯ Deutsch

◯ Français

Begrüssung

Liebe Kollegin, lieber Kollege

Hausarztmedizin ist die Basis unseres Gesundheitssystems. Zur Stärkung dieser Basis bietet die Stiftung zur Förderung der Weiterbildung in Hausarztmedizin (WHM) bzw. früher das Kollegium für Hausarztmedizin (KHM) seit 1998 Praxisassistenzen an.

Das Berner Institut für Hausarztmedizin BIHAM führt nun mit Unterstützung der Stiftung WHM eine Studie durch, um zu erfahren, was aus den ehemaligen PraxisassistenzärztInnen geworden ist. Auch interessiert uns, welche Eigenschaften der Praxisassistenz mit dem späteren Berufsbeginn als Hausarzt/-ärztin zusammenhängen.

Ihre Teilnahme an dieser Umfrage schätzen wir sehr!Ihre Angaben werden anonym ausgewertet. Die gesamte Studie benötigt zur Beantwortung einmalig 10-15 Minuten.

Sollten Sie selbst an den Resultaten interessiert sein, können Sie uns am Ende freiwillig Ihre E-Mail-Adresse bekannt geben.

Mit freundlichen Grüssen

Dr. med. Sven Streit
Studienleiter
Wissenschaftlicher Mitarbeiter
Berner Institut für Hausarztmedizin BIHAM
sven.streit@biham.unibe.ch

Persönliche Angaben

* Wir benötigen untenstehende Daten zur Verknüpfung mit den Angaben zu Ihrer Praxisassistenz in der Datenbank der WHM. Für die Auswertung werden nur anonymisiert Daten ausgewertet. Danke für Ihre Mithilfe!

Vorname	
Name	
E-Mail	

Praxistätigkeit

* Haben Sie die Praxistätigkeit als Hausarzt / Hausärztin in der Schweiz aufgenommen?

- ja
- nein

Angaben zu Praxistätigkeit

* Bitte machen Sie ein paar Angaben zu Ihrer Praxistätigkeit:

Beginn der Praxistätigkeit (Jahr)	
Postleitzahl der Praxis	
Arbeitspensum in %	

Praxisassistenz

* Wie wichtig war für Sie die Praxisassistenz in der Entscheidung Hausarzt/Hausärztin zu werden?

sehr unwichtig	unwichtig	neutral	wichtig	sehr wichtig
○	○	○	○	○

Gründe für Hausarztmedizin

Was waren Ihre drei wichtigsten Gründe Hausärztin bzw. Hausarzt zu werden?

1.

2.

3.

Berufswahl

* Welche Aussage in Bezug auf die Berufswahl trifft am ehesten auf Sie zu?

- ◯ Ich werde als Hausarzt /Hausärztin tätig sein, die Praxisaufnahme ist schon geplant
- ◯ Ich bin immer noch auf dem Track Hausarztmedizin, habe aber noch keine feste Planung zur Praxisaufnahme
- ◯ Ich bin unsicher, ob die Hausarztmedizin das Richtige für mich ist und konnte mich noch nicht entscheiden
- ◯ Ich habe mich für eine andere Tätigkeit als Hausarztmedizin entschieden
- ◯ Keine der obigen Antworten trifft auf mich zu, sondern:

Geplante Praxistätigkeit

* Bitte machen Sie ein paar Angaben zu Ihrer geplanten Praxistätigkeit:

Voraussichtlicher Beginn der Praxistätigkeit (Jahr)	
Postleitzahl der Praxis	
Voraussichtliches Arbeitspensum in %	

Andere Tätigkeit

Für welche Tätigkeit haben Sie sich entschieden?

Was trifft für Sie zu?

- ◯ Ich halte es für nicht ausgeschlossen, dass ich später noch in die Hausarztmedizin wechsle
- ◯ Ich denke nicht, dass ich später in die Hausarztmedizin wechseln werde
- ◯ anderes / Kommentar

Gründe gegen die Hausarztmedizin

Was waren Ihre drei wichtigsten Gründe nicht Hausärztin bzw. Hausarzt zu werden?

1.

2.

3.

Accueil

Chers/chères collègues,

comme nous le savons, la médecine de famille est la base de notre système de santé. Afin de consolider cette base, la FMF (Fondation pour la promotion de la formation en médecine de famille), respectivement auparavant le Collège pour la médecine de premier recours (Kollegium für Hausarztmedizin KHM), offre depuis 1998 des postes d'assistants en cabinet.

Avec le soutien de la FMF, le BIHAM (Institut bernois pour la médecine de famille) initie une étude afin de savoir ce que les anciens assistants en cabinet sont devenus. De plus, nous sommes intéressés à la question de savoir quel aspect de l'assistanat en cabinet a pu jouer un rôle dans l'engagement ultérieur comme médecin de famille.

Nous apprécions beaucoup votre participation à cette enquête. Vos réponses seront traitées sous le couvert de l'anonymat. La participation ne prend en tout qu'entre10 à 15 minutes en une seule fois.

Si vous êtes intéressés à connaître les résultats de l'enquête, nous vous invitons d'indiquer, à titre facultatif, votre adresse mail à la fin de ce questionnaire.

Dr. med. Sven Streit
directeur du projet
collaborateur scientifique BIHAM
sven.streit@biham.unibe.ch

Renseignements personnels

* Nous avons besoin de ces renseignements afin de pouvoir créer un lien vers les informations concernant votre assistanat en cabinet, contenues dans la banque de données de la WHM. Dans le cadre de cette enquête, les données sont traitées sous couvert d'anonymat. Merci de votre collaboration!

Prénom

Nom

E-Mail

Activité en cabinet

* Avez-vous commencé une activité de médecin de famille, en cabinet?

- ◯ oui
- ◯ non

Indications concernant l'activité en cabinet

* Veuillez nous donner quelques indications concernant votre activité en cabinet:

Début de l'activité (année)	
Case postale du cabinet	
Taux d'activité en %	

Assistanat en cabinet

* Lors de votre décision de devenir médecin de famille, quel rôle a joué votre assistanat en cabinet?

pas du tout important	peu important	neutre	important	très important
○	○	○	○	○

Raisons militant en faveur de la médecine de famille

Quelles étaient pour vous les trois raisons les plus importantes de devenir médecin de famille?

1.

2.

3.

Choix de profession

* Quelle affirmation concernant le choix de la profession s'applique le mieux à votre cas?

- ◯ Je serai médecin de famille, l'ouverture du cabinet est déjà planifiée
- ◯ Je suis toujours sur le track médecine de famille, mais n'ai pas encore de projet précis concernant l'ouverture d'un cabinet
- ◯ Je ne suis pas sûr que la médecine de famille soit le bon choix pour moi et n'ai pas encore pu prendre une décision
- ◯ Je me suis décidé pour une activité autre que la médecine de famille
- ◯ Aucune des affirmations ci-dessus s'applique à moi, mais plutôt:

Projet d'activité en cabinet

* Veuillez nous donner quelques indications concernant votre projet d'activité en cabinet:

Début probable de l'activité (année)	
Case postale du cabinet	
Taux d'activité prévu	

Raisons militant contre la médecine de famille

Quelles étaient les trois raisons les plus importantes pour votre décision de ne pas devenir médecin de famille?

1.

2.

3.

Autre activité

Quelle activité avez-vous décidé de choisir?

Quelle affirmation s'applique-t-elle à vous:

- Je considère qu'il n'est pas exclu que je me décide ultérieurement pour la médecine de famille
- Je ne pense pas que je me déciderai plus tard pour la médecine de famille
- Autre / commentaire

Kommentare / Commentaires

Haben Sie noch Anmerkungen oder Kommentare?
Avez-vous des remarques ou des commentaires?

Geben Sie erneut Ihre Emailadresse an, wenn Sie über die Umfrageresultate informiert werden möchten.
Veuillez nous indiquer votre adresse de mail pour le cas où vous aimeriez être tenu au courant des résultats du sondage.

Herzlichen Dank! Sie haben es geschafft.
Durch drücken auf "Umfrage abschliessen" ist Ihre Teilnahme abgeschlossen.

Un grand merci! Vous l'avez fait!
Pour terminer la séance, veuillez appuyer sur le bouton "terminer le sondage"
