# Supplementary material for: Optional part-time and longer GP training modules in GP practices associated with more trainees becoming GPs – a cohort study in Switzerland
Source: BMC Fam Pract. 2018 Jan 5;19:5. doi: 10.1186/s12875-017-0706-1 (PMC5756440; doi:10.1186/s12875-017-0706-1)
Supplement: Supplementary file 2 — German and French version of the survey used. (PDF 266 kb) [file 12875_2017_706_MOESM2_ESM.pdf]

UNIVERSITÄT BERN

WHM FMF

Stiftung zur Förderung der Weiterbildung in Hausarztmedizin
Fondation pour la Promotion de la Formation en Médecine de Famille
Fondazione per la Promozione della Formazione in Medicina di Famiglia

From training to profession - becoming a General Practitioner

Dear colleagues,
Primary Medicine is the base of our health system. Since 1998, the Stiftung zur Förderung der Weiterbildung in Hausarztmedizin, WHM (Foundation for the Promotion of Family Medicine) offers opportunities to participate in General Practitioner (GP) trainings to strenghten this base.
The Bernese Institute for Family Medicine, supplied by the WHM, surveys those who took advantage of this program, in part to determine which aspects of the training are associated with the decision to become a General Practitioner or join a GP in practice.
We appreciate your participation in this survey. Your data will remain anonymous. You will need 10-15 minutes to fill out the survey.
If you are personally interested in the results, please write your e-mail address at the end of the survey and we will keep you informed.

Kind regards,

Dr. med. Sven Streit
Studienleiter
Wissenschaftlicher Mitarbeiter
Berner Institut für Hausarztmedizin BIHAM
sven.streit@biham.unibe.ch

Personal data
We need your personal data so we can link your survey answers to the evaluation form you filled out after your GP training (database WHM).

First name:____________________
Last name:____________________
E-mail address:____________________

Survey

1. Are you currently working in a GP office as a GP?

   ☐ yes -> Please continue with question #2.
   ☐ no -> Please continue with question #5.

2. Please tell us about your work:

   Date you started working in a GP office: ____________________
   Postal Code of your GP office: ____________________
   What percentage to do you work (e.g., 50%): ____________________

3. Did your GP training play an important role in your decision to become a GP?

   ☐ very unimportant
   ☐ unimportant
   ☐ neutral
   ☐ important
   ☐ very important

4. List the three most important reasons you became a GP.

   1. ____________________
   2. ____________________
   3. ____________________

Please continue with question #10.

5. Which of the following statements best fits your current professional path?

☐ I will definitely work as a GP, and have made my plans to work in a GP office.
☐ I am still on the Family Medicine track, but I don't yet have a fixed plan for starting working in a GP office
☐ I am not sure if Family Medicine is right for me, and I cannot not make a decision yet.
☐ I decided against Family Medicine.
☐ Another option (please describe): I ________________________________

6. Please tell us about your plans to work in a GP office:

Expected start date:________________________________
Where do you plan to open your office? (postal code):________________________________
What percentage to do you plan work (e.g. 50%): ________________________________

7. Which career track did you decide to follow?

____

8. Which of the following statements is true for you?

☐ I might switch to Family Medicine
☐ I don't think I will switch to Family Medicine
☐ Other comments:

____

9. List the three most important reasons you decided against becoming a GP.

1. ________________________________
2. ________________________________
3. ________________________________

10. Do you have any other comment?

____

11. Please enter your e-mail address again, if want you to receive the results of the study.

____

Thank you very much for your participation!

Please return this survey to:
Post: Berner Institut für Hausarztmedizin, BIHAM zHd. Sven Streit, Gesellschaftsstrasse 49, 3012 Bern
Fax: 031 632 89 90
